# Spatial heterogeneity of radiolabeled choline positron emission tomography in tumors of patients with non-small cell lung cancer: first-in-patient evaluation of [^18^F]fluoromethyl-(1,2-^2^H_4_)-choline

**DOI:** 10.7150/thno.47298

**Published:** 2020-07-09

**Authors:** Suraiya Dubash, Marianna Inglese, Francesco Mauri, Kasia Kozlowski, Pritesh Trivedi, Mubarik Arshad, Amarnath Challapalli, Tara Barwick, Adil Al-Nahhas, Rex Stanbridge, Conrad Lewanski, Matthew Berry, Frances Bowen, Eric O. Aboagye

**Affiliations:** 1 Department of Surgery and Cancer, Imperial College London, United Kingdom.; 2 Department of Radiology/Nuclear Medicine, Imperial College Healthcare NHS Trust, London, United Kingdom.; 3 Department of Surgery and Cancer, Imperial College Healthcare NHS Trust, London, United Kingdom.; 4 Department of Medicine and Integrated Care, Imperial College Healthcare NHS Trust, London, United Kingdom.

**Keywords:** [^18^F] Fluoromethyl-(1, 2-^2^H_4_)-choline, Positron Emission Tomography, heterogeneity choline metabolism, choline kinase alpha, lung cancer.

## Abstract

**Purpose**: The spatio-molecular distribution of choline and its metabolites in tumors is highly heterogeneous. Due to regulation of choline metabolism by hypoxic transcriptional signaling and other survival factors, we envisage that detection of such heterogeneity in patient tumors could provide the basis for advanced localized therapy. However, non-invasive methods to assess this phenomenon in patients are limited. We investigated such heterogeneity in Non-Small Cell Lung Cancer (NSCLC) with [^18^F]fluoromethyl-(1,2-^2^H_4_) choline ([^18^F]D4-FCH) and positron emission tomography/computed tomography (PET/CT).

**Experimental design**: [^18^F]D4-FCH (300.5±72.9MBq [147.60-363.6MBq]) was administered intravenously to 17 newly diagnosed NSCLC patients. PET/CT scans were acquired concurrently with radioactive blood sampling to permit mathematical modelling of blood-tissue transcellular rate constants. Comparisons were made with biopsy-derived choline kinase-α (CHKα) expression and diagnostic [^18^F]fluorodeoxyglucose ([^18^F]FDG) scans.

**Results**: Oxidation of [^18^F]D4-FCH to [^18^F]D4-fluorobetaine was suppressed (48.58±0.31% parent at 60 min) likely due to the deuterium isotope effect embodied within the design of the radiotracer. Early (5 min) and late (60 min) images showed specific uptake of tracer in all 51 lesions (tumors, lymph nodes and metastases) from 17 patients analyzed. [^18^F]D4-FCH-derived uptake (SUV_60max_) in index primary lesions (n=17) ranged between 2.87-10.13; lower than that of [^18^F]FDG PET [6.89-22.64]. Mathematical modelling demonstrated net irreversible uptake of [^18^F]D4-FCH at steady-state, and parametric mapping of the entire tumor showed large intratumorally heterogeneity in radiotracer retention, which is likely to have influenced correlations with biopsy-derived CHKα expression.

**Conclusions**: [^18^F]D4-FCH is detectable in NSCLC with large intratumorally heterogeneity, which could be exploited in the future for targeting localized therapy.

## Introduction

Choline radiotracers, including [^11^C]choline, [^18^F]fluoromethylcholine ([^18^F]FCH) and [^18^F]fluoroethylcholine ([^18^F]FEC) are approved for use in detecting recurrent prostate cancer in patients with rising PSA despite having received definitive treatment together with investigational use in cancers of the brain, lung, liver and bladder 1. Evolving literature also suggests potential utility in response monitoring 2. Tumor PET images represent transport, phosphorylation and retention of choline metabolites in tumor cells 3. [^11^C]choline and [^18^F]FCH are rapidly oxidized to [^11^C]betaine/[^18^F]fluorobetaine by choline oxidase mainly in kidney and liver tissues, with metabolites detectable in plasma soon after injection of the radiotracer 4-7. To preclude interpretation of complex imaging signals, we developed a more metabolically stable fluorocholine analogue, [^18^F]fluoromethyl-(1, 2-^2^H_4_)choline ([^18^F]D4-FCH) based on the deuterium isotope effect 6, 8, 9.

Radiotherapy, alone or in combination with systemic therapies are the mainstay of treatment in patients diagnosed with stage I-IV NSCLC, and with advances in radiotherapy planning and delivery, a remarkable improvement in local tumor control has been seen 10, 11. It is hypothesized that imaging approaches for detecting the multiple factors that influence radioresistance would make further impact. For instance, hypoxia/hypoxia signaling is known to remarkably influence radioresistance, which has led to investigation of pre-therapy [^18^F]fluoromisonidazole PET ([^18^F]FMISO PET) to identify aggressive hypoxic components of tumors which can be targeted with a radiation boost with aim of reducing recurrence 12. In view of logistic issues with the latter e.g. 2-3 h to allow for (a relatively low) imaging contrast, other strategies are warranted. Choline transport and metabolism are upregulated in cancer cells and serve as potential targets in PET imaging using radiolabeled choline tracers. Choline-related metabolites including phosphocholine display a highly heterogeneous spatio-molecular distribution in tumors by high resolution mass spectrometry imaging, and expression of choline kinase-α (CHKα) is regulated transcriptionally by hypoxia 13. In early-stage NSCLC CHKα mRNA expression is prognostic indicating that aspects of tumor aggressiveness regulate the heterogeneous choline metabolic phenotype 14. The most widely used radiotracer for tumor imaging is [^18^F]fluorodeoxyglucose ([^18^F]FDG) which measures glucose utilization. [^18^F]FDG does not overlap with hypoxia measured by [^18^F]FMISO PET at the global or voxel level^15^.

Having developed [^18^F]D4-FCH for late (60 min) imaging of choline transport and phosphorylation in pre-clinical models and demonstrated safety/appropriate organ dosimetry in healthy volunteers, the objective of this study was to investigate imaging characteristics of the tracer in tumors and metastases, assess tumor heterogeneity and further confirm whether simplified measures of radiotracer uptake were associated with more complex kinetic variables so as to support use of such simplified measures; particular for standard late (60 min) imaging where, for [^11^C]choline and [^18^F]FCH, parent radiotracer levels in blood are either very low or undetectable. The setting for this first human study is non-small cell lung cancer (NSCLC). The choice is predicated on previous work suggesting that CHKα expression correlates with survival 14; and routine use of [^18^F]FDG PET in this setting, to enable comparison.

## Experimental design

### Patients

A prospective non-randomized feasibility study was conducted in patients with newly diagnosed localized, locally advanced or metastatic NSCLC who were fit for surgery, radiotherapy or systemic therapy. Patients with histologically confirmed NSCLC were recruited from Respiratory and Oncology clinics at Imperial College NHS Healthcare Trust over a period of 3 years (2014-2017). All patients included were ≥18 yr., good performance status (ECOG ≥2), and had clinically acceptable blood parameters. All had at least one measurable site of disease ≥ 2cm (primary tumor or lymph node). Excluded from study were patients participating in other clinical trials with investigational drugs within 30 days prior to study entry, pregnant or lactating women and patients diagnosed with an invasive malignancy within the last 5 years (excluding basal cell carcinoma). Ethical approval was granted for this study by the West London and GTAC NRES committee, and the Medicine and Healthcare Products Regulatory Agency (MHRA). All patients provided written informed consent in accordance with the Declaration of Helsinki and the administration of radioactivity was approved by the Administration of Radioactive Substances Advisory Committee (ARSAC).

### Radiopharmaceutical preparation, image acquisition and analysis

[^18^F]D4-FCH was synthesized from its precursor as previously described by our group, 2011 16, 17**.** As part of standard investigations, patients underwent routine imaging with contrast enhanced computed tomography (CECT) of chest and upper abdomen and a staging [^18^F]FDG PET/CT. [^18^F]D4-FCH PET/CT was performed after routine standard investigations and prior to any therapy. Images were acquired on a Siemens Biograph 6 TruePoint PET/CT scanner (with TrueV; extended field of view) with 21.6-cm axial and 60.5-cm transaxial fields of view. A thoracic CT scan (130 kV; exposure, 30 effective mAs; pitch, 1.5; slice thickness, 5 mm; rotation time, 0.6 s; resulting in an effective dose of 2.5 mSv) was performed for attenuation correction and anatomical localization. This was then followed by tracer injection (a maximum dose of 370MBq of [^18^F]D4-FCH) and dynamic imaging (single-bed position over thorax and mediastinum) for 66 min followed by a half-body static (mid-thigh to vertex) attenuation CT and PET scan (3 min per bed position). Discrete venous bloods and plasma (at 5, 10, 15, 30, 60 min post injection) were obtained for radioactivity counting and metabolite analysis, as previously described 16, 17 and applied in oncological studies 18, 19. The time course of metabolite and parent fraction measured directly by HPLC (experimental data) for D4 Choline are summarized in Figure S1.

All dynamic [^18^F]D4-FCH PET/CT data were reconstructed using the ordered-subsets expectation maximization algorithm (OSEM - 3 iterations and 21 subsets) with corrections for dead time, scatter, attenuation, and radioactive decay. For each patient, volumes of interest (VOIs) for both [^18^F]D4-FCH and [^18^F]FDG PET/ CT were generated using Hermes (Hermes Diagnostics, Stockholm, Sweden) by a single investigator (SD) onto fused image datasets (PET/CT). Volumes of interests (VOIs) were drawn manually over the whole tumor, in normal background organs (2 cm fixed spheres in normal lung tissue, vertebral body and muscle), in the aorta (1cm fixed sphere) and in lymph nodes, when present. An additional VOI was drawn around the aorta (1cm fixed sphere) for an image derived calculation of the arterial input function. The parent plasma input function was generated by normalizing to plasma-over-blood ratio and correcting for metabolites. In addition to that, to account for the presence of labelled metabolites, the metabolite time activity curve in plasma was used as input of the double input spectral analysis 20. PET data was quantified with the semi-quantitative Standardized Uptake Value (SUV) and tracer kinetics was studied with quantitative methods: spectral, graphical and compartmental analyses. The work by Boellaard's lab for [^18^F]FCH 21 indicated discordance between SUV and kinetic variables, which warranted investigation of tracer kinetics for [^18^F]D4-FCH. PET data were quantified with the semi-quantitative Standardized Uptake Value (SUV) and tracer kinetics were studied with quantitative methods: spectral, graphical and compartmental analyses. The analysis started with the implementation of Spectral Analysis (SA) which, without any a priori assumption on the kinetics of the tracer, provides a “kinetic spectrum” representing the functional processes in which the tracer is involved. Hence, from this spectrum it is possible to obtain a complete description of tracer kinetics. It is also possible to identify the number and the type of compartments necessary for the data modelling- necessary information for the following graphical and compartmental analyses.

SUV: PET quantification is routinely performed using the SUV, a semi-quantitative index computed as the raw image counts normalized by the injected dose, and generally, the patient's body weight. From SUV tissue radioactivity curves, we evaluated mean and maximum values of SUV and areas under the SUV curve (SUV_AUC_; from zero to time *t*) at 5, 30 and 60 min (SUV_5mean_, SUV_30mean,_ SUV_60mean_, SUV_5max_, SUV_30max,_ SUV_60max_, SUV_AUC5mean_, SUV_AUC30mean,_ SUV_AUC60mean_, SUV_AUC5max_, SUV_AUC30max,_ SUV_AUC60max_). We also evaluated mean and maximum SUV for the later static [^18^F]D4-FCH scan (SUV_60mean_s_, SUV_60max_s_).

Spectral analysis (SA): Without any a priori assumption on the kinetics of the tracer, SA provided the K_1_ (K_1__SA), the influx rate constant, and the K_i_ (K_i__SA), net uptake of tracer in tissue. Spectral analysis provided also the impulse response function (IRF) of the compartmental models used in PET, evaluated as an analytical sum of exponentials 22. SA was conducted for each patient choosing a grid of 100 components in the range of (0.0001, 1) 23, 24. A double input SA (DI-SA) was also implemented to account for the contribution of the radiometabolites. This approach is based on the method implemented by Tomasi et al. 20, 25.

Graphical analysis: Graphical methods are the simplest approaches for dynamic PET data quantification as they are based on a linearization of compartmental model's differential equations. *Patlak plot* was used to estimate K_i_ (K_i__P), the net irreversible uptake rate constant, which quantifies the rate at which the tracer is irreversibly trapped 26. The *Logan plot* is the counterpart of the Patlak plot for reversible radiotracers. This method was used to estimate the single macroparameter V_t_ (V_t__L), the total volume of distribution of the tracer in the tissue 27.

Compartmental analysis: To investigate the best kinetic model, methods by Fan and colleagues 28, were employed with the MICK software (modelling-Input-function-Compartmental-Kinetics) and Matlab (Mathworks, R2015b). Three different kinetic models were tested: the reversible 1-tissue 2k (1TCM2k) model, the 2-tissue 3k (2TCM4k) model, and the irreversible 2-tissue 3k model (for which k implies the rate constant for tracer for different kinetic compartments).

Parametric maps and distribution kernels: A Kernel function was used to model the voxel distribution profiles of each parameter and to obtain skewness, kurtosis and ratio (skewness/kurtosis) values for each parameter.

### Statistical analyses

The model providing the best fits to the lesion time-activity curves was selected on the basis of the Akaike information criterion (AIC) 29:


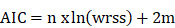
 (Equation 1)

where *m* denotes number of parameters, *n* equals the sum of degrees of freedom and number of parameters, and *wrss* denotes weighted residual sum of squares 30.

The Kolmogorov-Smirnov test was used to assess the normal distribution of each parameter distribution. Differences among tumor and healthy tissues were tested with the Wilcoxon Sum Rank test and corrected for multiple comparisons using Bonferroni approach. Spearman's rank test was used to verify the correlation between parameters. All statistical tests were run in Matlab (Mathworks, R2018b).

### CHKα Immunohistochemistry

CHKα immunohistochemistry was conducted as previously described studies using a primary polyclonal, human anti-CHKα antibody as per manufacturer's instructions (Sigma-Aldrich, HPA024153,1:20 dilution); the intensity of cytoplasmic and nuclear staining were scored (score 1: low intensity, 2: moderate and 3: high) 31, 32. The relationship between PET uptake parameters (SUV_60mean_ and SUV_60max_) and CHKα expression, determined by immunohistochemistry on lung tumor tissue and lymph node specimens, was established. In addition, commercially available tissue microarrays (TMAs; US Biomax Inc.) were obtained (HLug-Ade090Lym-01; HLug-Squ090Lym-01; HLug-Ade150Sur-01; HLug-Squ150Sur-01) and staining performed as above.

## Results

### Patients

Seventeen patients with confirmed NSCLC were enrolled to the study. All patients fulfilled the inclusion criteria and provided written informed consent. Patient characteristics and primary treatment modalities are shown (Table 1). The mean age ± sd were 64 ± 11y; age range 39-84 y; weight ± sd, 68.9 ± 15kg; weight range, 49.7-93.3 kg), respectively. Nine patients were diagnosed with squamous cell carcinoma and 8 had adenocarcinoma. Twelve out of 17 patients had nodal metastases diagnosed on mediastinoscopy and confirmed as positive on tumor and nodal resection on surgical histopathology. Five patients eligible for surgery underwent a lobectomy or pneumonectomy, or wedge resection with hilar and mediastinal lymph node sampling, or en bloc resection. Primary systemic treatment consisted of platinum doublet chemotherapy (gemcitabine and carboplatin, pemetrexed and cisplatin or carboplatin). Other treatment options included radical radiotherapy, chemoradiotherapy, and palliative high-dose radiotherapy. Treatment decisions were made by the lung cancer multidisciplinary team. Of the adenocarcinoma patients tested, 6/6 were negative for EGFR (epidermal growth factor receptor) mutation, 2/3 were ALK (Anaplastic lymphoma kinase gene) positive, and 1/5 were KRAS (Kristen rat sarcoma viral oncogene) mutant. Median follow-up for all patients was 15 months (range 3-47 months). Eleven patients out of 17 died of lung cancer at time of last follow-up. Local and distant recurrence were identified in 9 patients.

### Radiopharmaceutical and *in vivo* radiotracer stability

**Figure 1:**
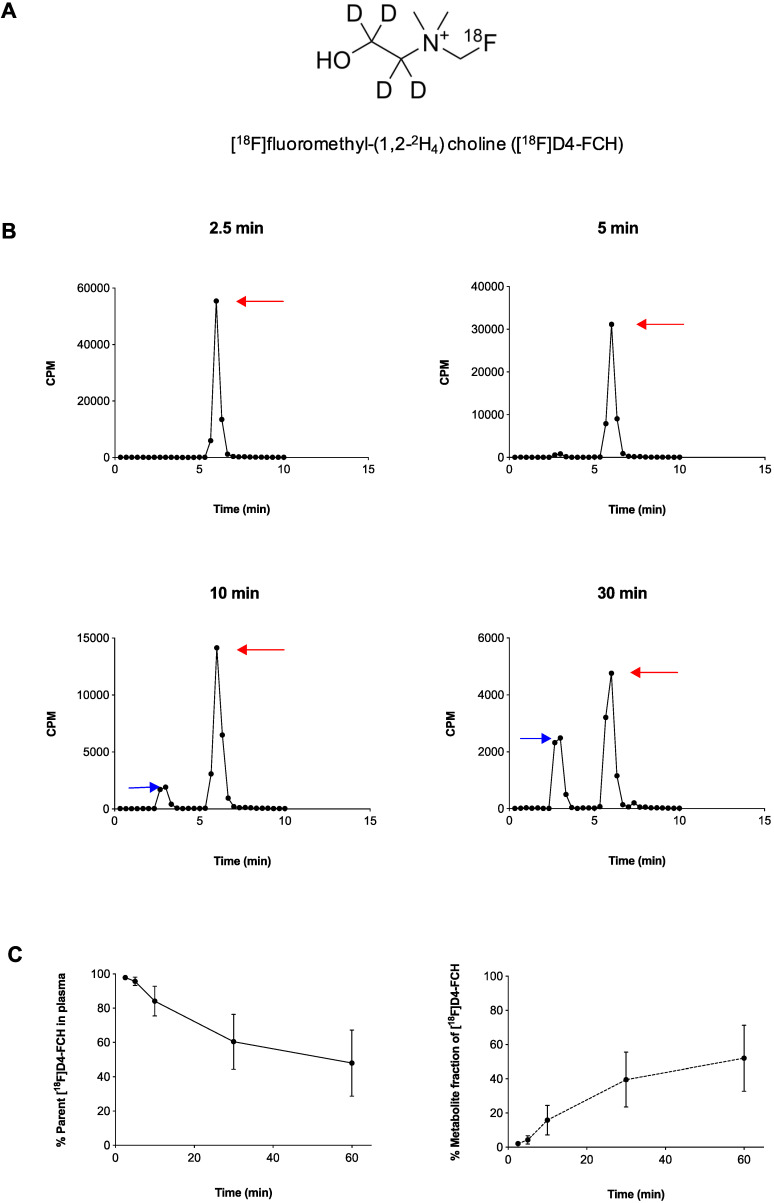
** Structure and metabolic stability of [^18^F]D4-FCH. (A)** Chemical structure of [^18^F]fluoromethyl-(1,2-^2^H_4_) choline ([^18^F]D4-FCH). **(B)** Typical high-performance liquid chromatogram (HPLC) of [^18^F]D4- FCH in plasma at 2.5, 5, 10 and 30 min time-points in a patient. Red arrows point to parent tracer and blue arrows point to metabolite peak (betaine) of [^18^F]D4-FCH. Y-axis scale adjusted to highlight metabolite peak. CPM = counts per minute. **(C)** Mean percentage parent tracer and metabolite fraction over time (min).

Radiochemical purity of [^18^F]D4-FCH was 100% on completion of synthesis with a mean (± sd) specific activity of 61 ± 76 GBq/μmol (range, 21-353 GBq/μmol) and pH of 5.35 ± 0.45 (range 4.50 -5.81). The mean (± sd) dose injected in all patients was 300.5 ± 72. 9MBq (range 147.60 - 363.6MBq). [^18^F]D4-FCH was well tolerated by all patients with no immediate or delayed complications observed. As per healthy volunteers 17, the radiotracer was stable after injection into patients with only 48.6% of the parent converting mainly to a single metabolite ([^18^F]D4-fluorobetaine) after 60 min. Figure 1A shows the chemical structure of [^18^F]D4-FCH, the typical radio-high-performance liquid chromatograms (radio-HPLC, 1200 series system; Agilent) for a representative patient (Figure 1B), and the mean percentage metabolite and parent tracer fraction over time (Figure 1C).

### NSCLCs are avid for both choline and glucose pathway tracers

**Figure 2:**
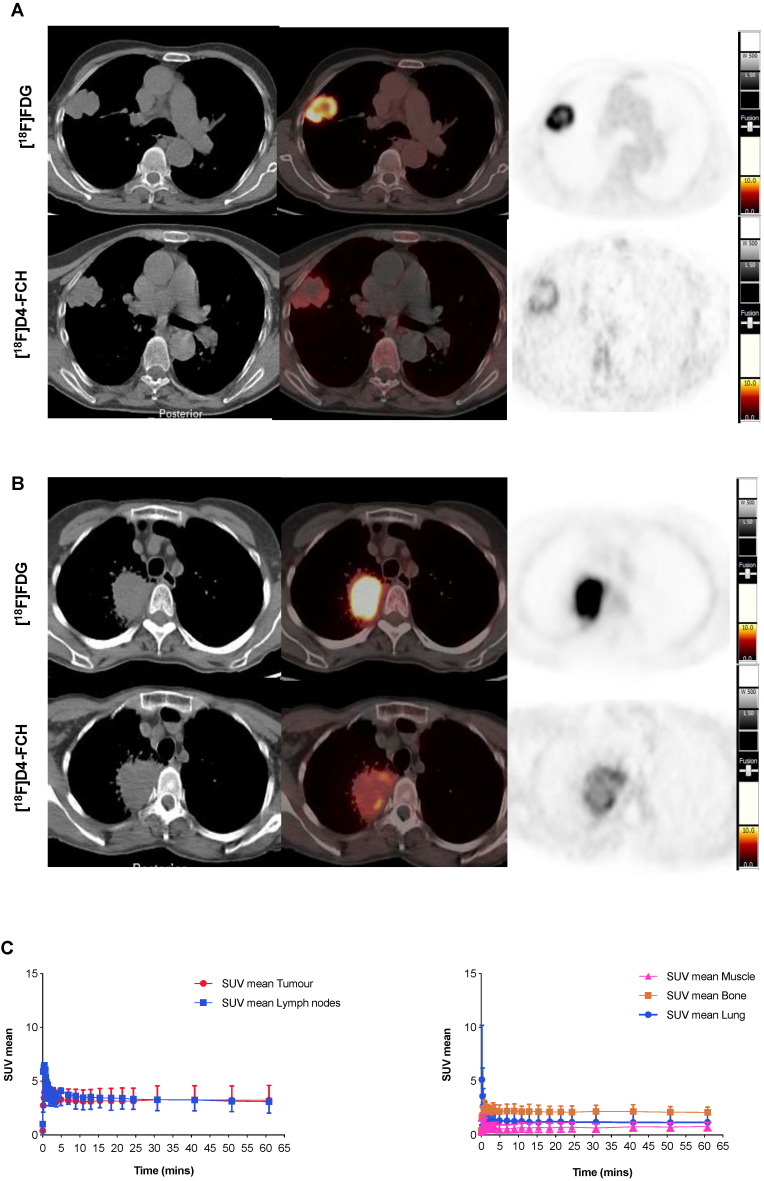
** [^18^F]D4-FCH and [^18^F]FDG Tissue uptake. (A)** Axial views on CT, fused PET/CT and PET for diagnostic [^18^F]FDG and [^18^F]D4-FCH in a patient with squamous cell carcinoma of the right lung (patient 3 - see Table 2); **(B)** similar images in a patient with an adenocarcinoma of the right lung (patient 13 - see Table 2), SUV intensity threshold 0-10; and; **(C)** Mean decay-corrected time-activity curves (TACs) for normal tissue (normal lung, bone, including bone marrow, and muscle) and tumor and lymph nodes in dynamic PET imaging.

Images acquired with [^18^F]D4-FCH PET, and comparison to [^18^F]FDG PET are shown (Figures 2A and B). Mean decay-corrected time activity curves (TACs) for tumor, lymph nodes and normal tissues are shown in Figure 2C (SUV at 5, 30 and 60 min were similar, Table 2; Table S1). Physiological uptake of [^18^F]D4-FCH PET was noted in the salivary glands, liver, kidneys, pancreas and bladder. All primary tumors and involved lymph nodes were visible on [^18^F]D4-FCH PET/CT. NSCLC lesions were avid for both [^18^F]D4-FCH PET and [^18^F]FDG in patients who underwent [^18^F]FDG PET/CT as part of their staging work-up. [^18^F]D4-FCH derived uptake (SUV_max_) in index primary lesions (n=17) ranged between 2.87 and 10.13; lower than that of [^18^F]FDG PET/CT (6.89 and 22.64) (Table 2; Table S1). On analysis of [^18^F]D4-FCH PET/CT (static) alone, the SUV_mean_ and SUV_max_ values were higher in the lymph nodes and metastases compared with primary tumor in 7 out of 17 patients. Corresponding nodal lesions on [^18^F]FDG PET/CT (where available) showed in general, higher SUV values but in 1 patient (patient 3), SUV was found to be lower than that of [^18^F]D4-FCH PET/CT. The noted [^18^F]D4-FCH PET uptake within the mediastinal lymph nodes was negative for malignancy on correlation with surgical histopathology. It is unclear whether relevant or not, but on further investigation this patient was noted to be a heavy smoker. Unlike prostate cancer where low level symmetrical [^18^F]FCH uptake is often seen in reactive nodes 33, this was not seen with [^18^F]D4-FCH PET. The same number of lesions were noted with [^18^F]FDG PET/CT apart from 1 patient where [^18^F]FDG demonstrated uptake of a lower vertebral metastasis (patient 6).

### Mathematical modelling of [^18^F]D4-FCH data support metabolic conversion to phosphocholine

**Figure 3:**
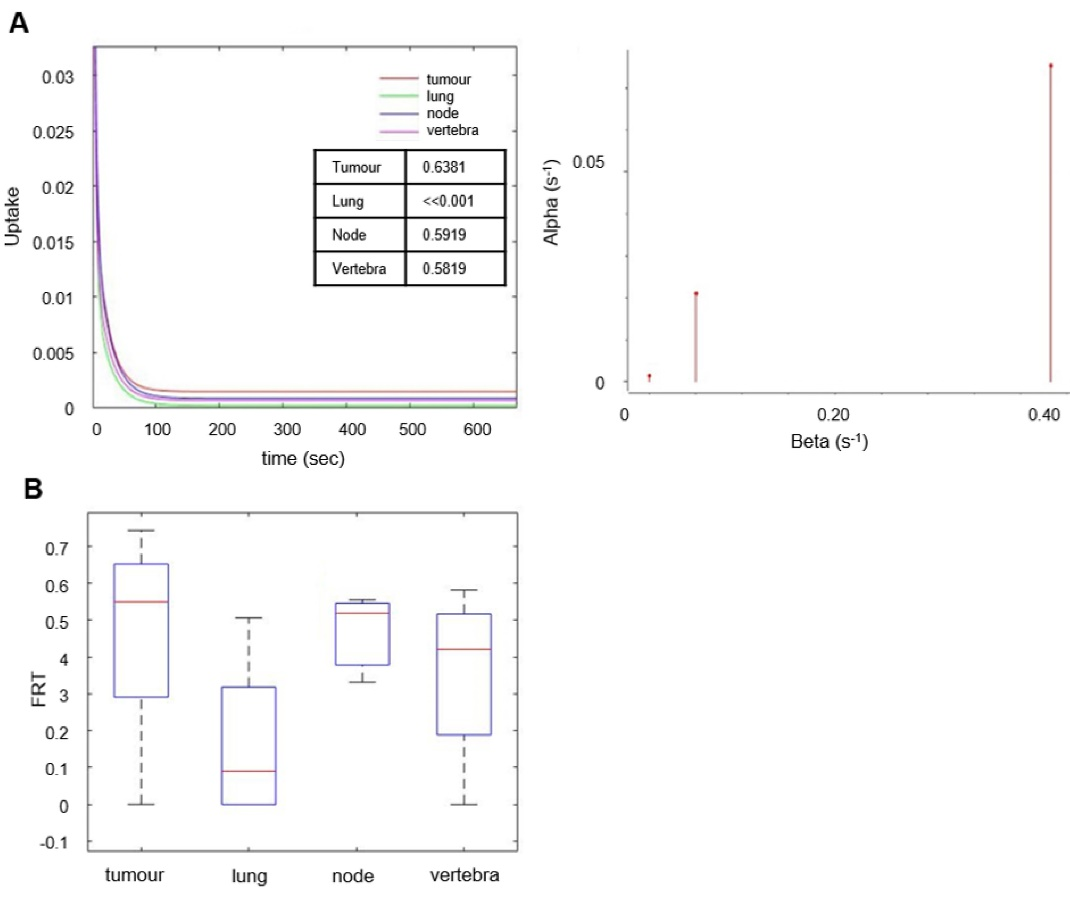
** Spectral analysis of [^18^F]D4-FCH in lung tumor. (A)** The unit impulse response function (IRF) of tumor compared with reference tissue (normal lung), lymph node metastases and vertebra (values of the fractional retention of the tracer (FRT) are shown) and spectrum of the kinetic components obtained using spectral analysis. **(B)** Box-and-whisker plots of FRT showing the highest retention component in tumor and lymph node metastases. Less retention was seen in the reference tissue (normal lung) and vertebra. Node refers to involved lymph node metastases.

The behavior of the tracer was investigated in a stepwise manner. With spectral analysis the kinetics of [^18^F]D4-FCH was described by a kinetic spectrum, which allowed the identification of the number and type of compartments necessary for the following graphical and compartmental analyses. On the basis of the AIC, standard and double input spectral analyses 25 were comparable (AIC of standard SA = 4.98 ± 34.76, AIC of DI-SA = 7.17 ± 34.61) with slightly better fits provided by DI-SA. SA revealed irreversible trapping component of [^18^F]D4-FCH in the tissue in 8 patients (Figure 3A, Figure S2). This was further confirmed by the fractional Retention of Tracer (FRT) calculated as IRF at 60 minutes relative to 1 min (p > 0.05) (Figure 3B) and by compartmental analysis (fitted to a two-tissue irreversible model - 2TCM3k - p < 0.05). Both Patlak and Logan plots are justified by the result of the spectral analysis. In fact, while the spectrum revealed a low frequency slow (irreversible) component, this did not occur in the entire cohort. SA revealed also a reversible trapping component in 3 patients. For this reason, both Patlak and Logan plot were implemented. The best fit was provided by Patlak plot (AIC = -57.00 ± 25.39), followed by Logan plot (AIC = 101.06 ± 37.22). Mean SA K_1_ (K_1__SA), SA K_i_ (K_i__SA), SA parent tracer volume of distribution (V_Tpar_), SA metabolite volume of distribution (V_Tmetab_), DI-SA K_i_, Patlak K_i_ (K_i__P), and Logan V_t_ (V_t__L) in primary tumors and healthy lung are summarized in Table 3. SA revealed irreversible trapping component of [^18^F]D4-FCH in the tissue in 8 patients (Figure 3A, Figure S2).

We previously demonstrated in mouse tumors that [^18^F]D4-FCH is phosphorylated to [^18^F]D4-phosphocholine or oxidized to [^18^F]D4-fluorobetaine, (with the latter occurring mainly in organs like liver or kidneys)^9^, ^25^. However, we did not conduct a head-to-head comparison between [^18^F]FCH and [^18^F]D4-FCH, and to date, only rodent studies have been conducted in a head-to-head manner. DI-SA was used to investigate the volumes of distribution of the parent and of the metabolite in the tissue. Table 3 shows the two volumes of distribution and the K_i__SA in primary tumors and lymph nodes. In most lesions with measurable trapping component, the main contributor to the distribution volume was the parent fraction; the higher contribution of metabolites to certain tumors with low tracer retention was also evident.

### Immunohistochemistry shows large variation of CHKα protein expression in lung tumors

**Figure 4:**
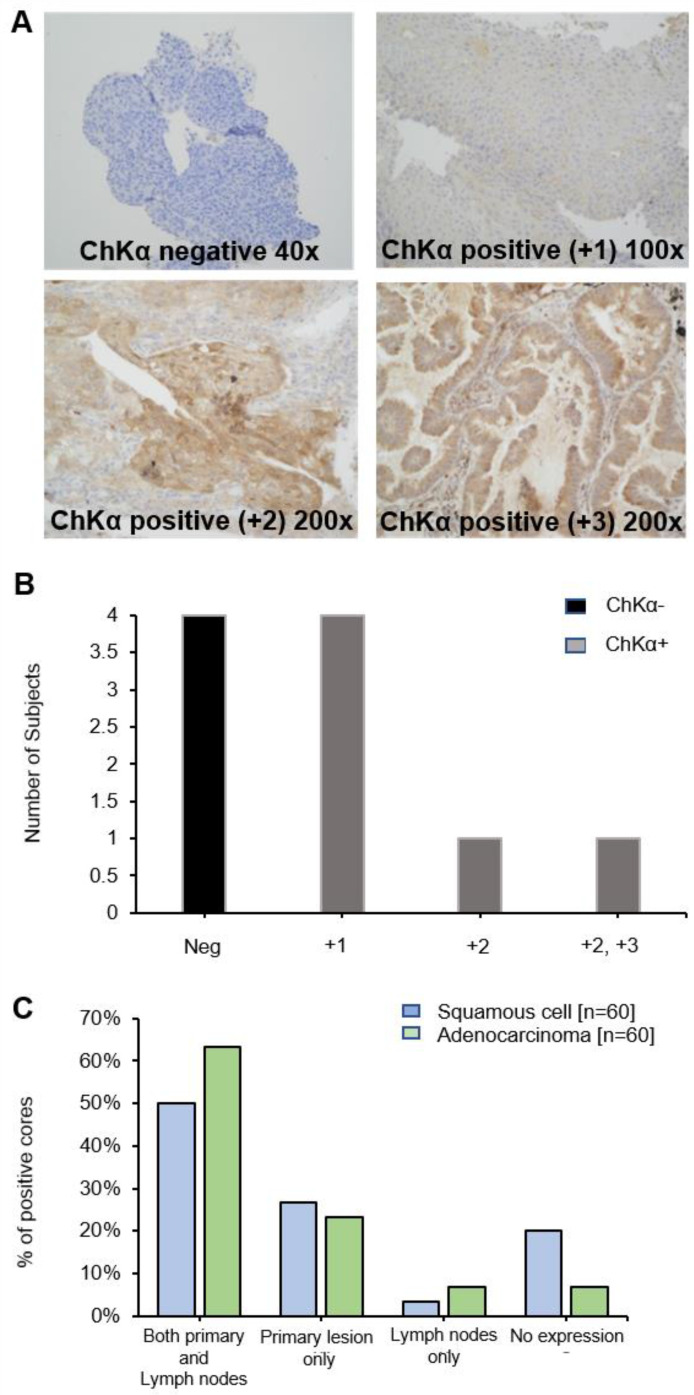
** Choline kinase alpha (CHKα) expression in NSCLC. (A)** Immunohistochemistry panel showing CHKα expression in lung tissue of patients with NSCLC. Examples are shown of CHKα negative, CHKα positive +1, +2 and +3 intensities on staining. Images are x200 magnification. **(B)** CHKα expression in lung tissue was found to be negative in four patients (Neg, patients 2, 10,12 and 14), +1 in four patients (patients 4, 5, 6 and 7), +2 in patient 3 and +2/+3 in patient 9. **(C)** Commercial TMAs (US Biomax Inc.): CHKα was expressed in both primary lesions and lymph nodes in 50% of squamous cell tissue and 63.3% of adenocarcinoma cores. 26.7% and 23.3% of cores expressed CHKα only in the primary squamous cell and adenocarcinoma tissue, respectively. 3.3% and 6.7% of cores expressed CHKα only in the squamous cell and adenocarcinoma lymph nodes, respectively. In both squamous and adenocarcinoma primary lesion and lymph nodes, no expression was found in 20% and 6.7% of cores, respectively.

Diagnostic biopsy tissues from primary tumor were available in 10 out of 17 patients, 6 of whom were positive for CHKα protein expression (Figure 4A and B) (lymph node biopsies were not available). No correlation was seen between PET uptake variables and CHKα protein expression, e.g., SUV_max_ and CHKα expression (*p* = 0.968). Of interest, two out of the 6 patients, who demonstrated higher [^18^F]D4-FCH uptake in lymph node metastases than primary tumor (patient 2 and 10), were negative for CHKα expression indicating tissue heterogeneity. To affirm that staining heterogeneity was not just pertinent to our prospective study, we investigated CHKα protein expression in commercially available TMAs (US Biomax Inc.). Sixty cores of squamous cell carcinoma and 60 cores of adenocarcinoma with no survival data, as well as 75 cores of each histopathology sub-type with corresponding survival data were available. Analysis of these tissue demonstrated substantial heterogeneity (~50% staining) of CHKα protein expression in lung lesions of both histologies (Figure 4C) supporting data from our prospective study. 48.95% of cores (35/69 squamous cell and 35/75 adenocarcinoma) showed cytoplasmic staining for CHKα. CHKα expression with staining intensity +2 was found in all T2a/b, N0/N1 and M0 cases. No correlation with survival and stage of disease was seen.

### Parametric maps of tumor [^18^F]D4-FCH PET show distinct spatial heterogeneity

**Figure 5:**
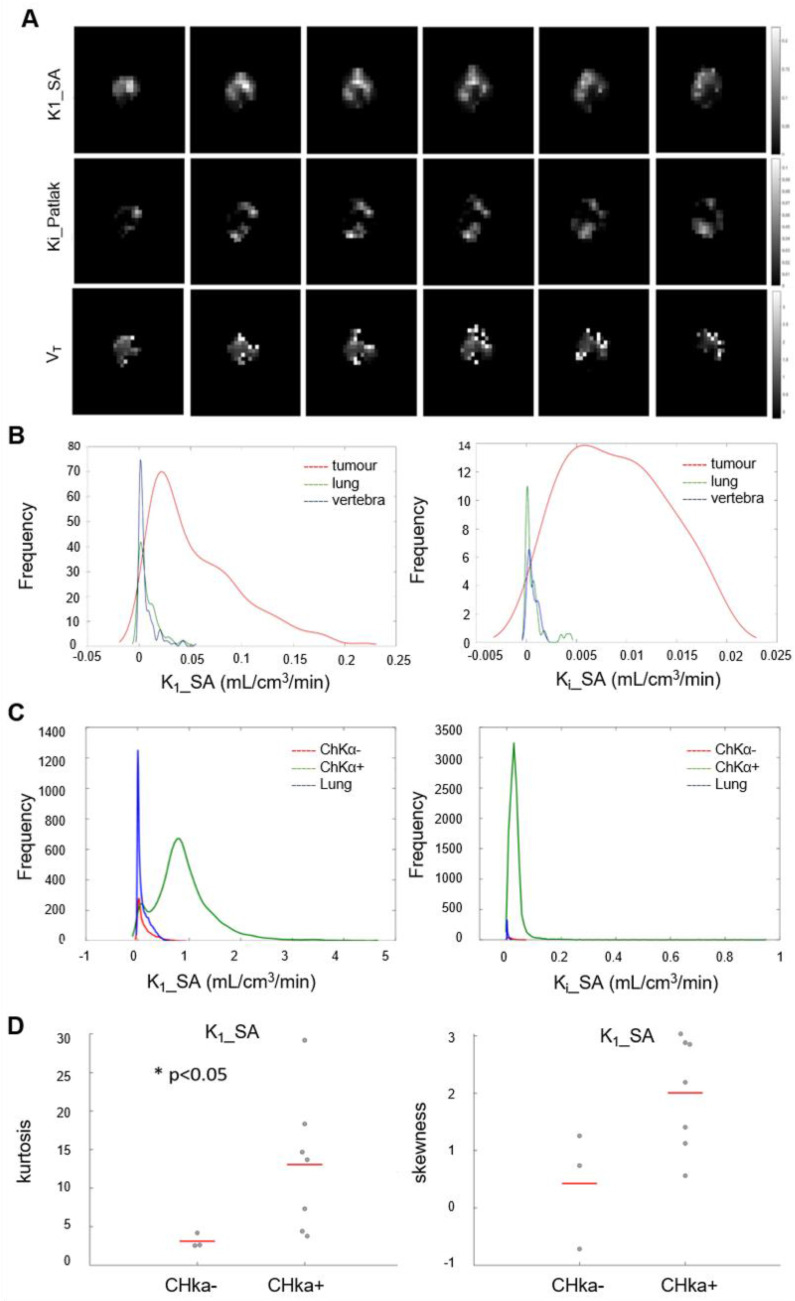
** Parametric analysis of [^18^F]D4-FCH. (A)** Spectral analysis at voxel level resulted with the K_1_ parametric map. Patlak and Logan methods resulted with 3D parametric maps of K_i_ and V_t_, respectively. **(B)** Kernel Distribution analysis of K_1__SA (on the left) and K_i__SA (on the right) of a representative patient. Tumor, reference tissue (normal lung) and vertebra are shown in this patient as an example. No lymph node metastases were present. On the y axis, the total number of data points per bin. The kernel distributions illustrate the heterogeneity of tumor tissue compared with normal tissue. Normal lung, shown in green, has a more homogenous peaked distribution, as expected. **(C)** Global kernel distributions of parametric maps (Spectral Analysis K_1_ on the left and SA_K_i_ on the right) evaluated in healthy lung (in blue, n = 10), lesions which showed a negative CHKα expression (in red, n = 3) and lesions which showed a positive CHKα expression (in green, n = 7). On the y axis, N is the total number of data points per bin. **(D)** Kurtosis and Skewness values of Spectral Analysis K_1_ map in patients with negative (n = 3) and positive (n = 7) CHKα expression. Statistically significant difference was found between the K_1__SA kurtosis of CHKα- and CHKα+ lesions (*p* < 0.05).

Initially we performed histogram analysis of 3D parametric maps of K_i__P, K_1__SA and V_t__L generated from Graphical and Spectral methods for tumor, normal lung (as reference tissue), lymph node metastases and vertebra (Figure 5A). Statistical differences between tumor and healthy tissues (entire volume-of-interest) were tested with the Wilcoxon rank sum test and were found to be significant (*p* < 0.05) for K_i__P, K_1__SA, K_i__SA and V_t__L. To interrogate spatial relationships, we constructed histograms of the data. Figure 5B shows the histogram analysis K_1__SA and K_i__SA for tumor, normal lung, lymph node metastases and vertebra in a representative patient. In the majority of analyzed cases, the voxel distribution profiles were non-Gaussian and modelling was performed with a Kernel function 34. From the results of distribution analysis, the flattened tumor distributions profiles (Figure 5B) testify the wider range of values covered by parametric maps (theoretically from higher skewness, lower kurtosis and/or higher skewness/kurtosis ratio relative to normal lung). Notably, a statistically significant lower kurtosis was seen for the K_1__SA kernel for CHKα negative tumors compared to CHKα positive tumors (Figure 5C and D; Table S2), indicating perhaps that one reason for CHKα negativity may be due to heterogenous sampling.

### Simplifying [^18^F]D4-FCH measures -comparison between SUV and pharmacokinetic parameters

The imaging protocol could be simplified using semi-quantitative methods instead of a full kinetic analysis. Results from SUV analyses and the quantification of dynamic PET data were compared. In particular, we compared K_i__P, K_1__SA, K_i__SA, K_i__CM and V_t__L to the mean and maximum SUV at 5 and 60 minutes ( SUV_5mean_, SUV_5max_, SUV_60mean_, SUV_60max_), and to the SUV_AUC_ mean and maximum evaluated at 5 and 60 minutes (SUV_AUC_5mean_, SUV_AUC_5max_, SUV_AUC_60mean_, SUC_AUC_60max_). Results of the linear regression analysis are summarized in Table S3. Commonly used simplified methods showed moderate correlation to K_i__P and K_1__SA (K_i__P vs. SUV_60mean_, R^2^ = 0.5308; K_i__P vs. SUV_AUC_60mean_, R^2^ = 0.5246; K_1__SA vs. SUV_60mean_, R^2^ = 0.4617; K_1__SA vs. SUV_AUC_60mean_, R^2^ = 0.4502). The lowest correlations resulted from the comparison between SUVs and K_i__SA, K_i__CM and V_t__L.

Because most patients had diagnostic [^18^F]FDG scans, we also assessed the correlation between [^18^F]D4-FCH and [^18^F]FDG SUVs (in the case of [^18^F]D4-FCH, both early - ~5 min and late ~60 min images from the dynamic scan). Correlations were weak when the two static scans (R^2^ = 0.1928 and R^2^ = 0.2478 for SUV_mean_ and SUV_max_, respectively) were compared, and only moderate when [^18^F]FDG SUVs were compared to [^18^F]D4-FCH SUVs evaluated from the dynamic scan (R^2^ = 0.4063 and R^2^ = 0.5080 for SUV_mean_ and SUV_max_, respectively).

## Discussion

The deuterium isotope effect involving reduced tunneling of choline substrates to the catabolic (choline oxidase) enzyme site has been proved in this study to stabilize [^18^F]D4-FCH in systemic circulation, thus permitting substrate transport and retention to be determined at early and late timepoints. By using [^18^F]D4-FCH PET/CT, we report that substantial heterogeneity for choline substrate transport and retention exists in lung tumors and might confound biopsy measurements of choline pathway protein expression.

This is the first study of [^18^F]D4-FCH in patients, having previously asserted safety and established dosimetry for human use 17. We confirmed the slow catabolism of [^18^F]D4-FCH to [^18^F]D4-fluorobetaine in patients using radio-HPLC. This is in contrast to [^11^C]choline and [^18^F]fluorocholine, which are rapidly catabolized 17, 21 leading to lower proportion of available substrate for tumor uptake for non-deuteriated analogues^35^, ^36^. Previous studies with choline tracers have attempted to overcome the rapid catabolism by scanning at 5-15 min. We demonstrated an ability to conduct scanning consistently at early (5 min) or late (60 min) timepoints; it is appreciated that differences in perfusion could affect interpretation of early scan protocols. Consequently, in all primary lung lesions uptake with [^18^F]D4-FCH PET/CT was demonstrated in this study. Using mathematical modelling we were able to interpret the trapping of [^18^F]D4-FCH as contributed by substrate transport and conversion to phosphocholine.

The availability of routine [^18^F]FDG PET/CT scans in 12 of the 17 patients allowed us to establish to what extent the two tracers were used by lung tumors. While lesion detection was comparable between the two tracers, uptake intensity (SUV) was overall higher with [^18^F]FDG (2.87-10.13 vs 6.89-22.64). Studies in lung cancer using [^11^C]choline PET, have shown variable results. When comparing [^11^C]choline, typically evaluated at 5-15 min, and [^18^F]FDG, typically evaluated at 60 min for detection of mediastinal lymph node metastases, Hara et al reported excellent performance (100% sensitivity for both tracers in 29 patients) prior to thoracotomy and ipsilateral node dissection 37. Other studies have reported a lower diagnostic sensitivity for [^11^C]choline compared to [^18^F]FDG PET in detecting nodal disease, but superior sensitivity for detecting of metastatic disease^38^, ^39^. However, it is important to note that the above studies were PET-only (rather than PET/CT), and in the study by Hara and colleagues, sensitivity may have been overestimated by the low SUV criteria used for lesion detection 37.

The high spatio-molecular distribution of choline metabolites in mouse tumors 13, and aforementioned resistance phenotype regulating/associated with choline metabolism supports exploitation of this radiotracer for localized therapy planning. Choline tracers have indeed been used to assess target volume, support focal dose escalation decisions and for monitoring response to external beam radiotherapy 40, particularly in the setting of radical prostate radiotherapy with concurrent androgen deprivation 41. Thus, we investigated whether similar spatio-molecular distribution of choline metabolites exists and found that radiotracer transfer from blood to tissue, (described by K_1_) and radiotracer retention (described by K_i_; mainly due to labelled phosphocholine formation) were highly heterogeneous in distribution. This provides the opportunity to test in a prospective manner, the role of [^18^F]D4-FCH PET/CT for planning focal dose escalation to “highest-risk” tumor regions.

Increased CHKα expression has been described in several tumor types 42-44. The role of CHKα mRNA in malignancy and clinical relevance as a prognostic factor in early-stage lung cancer has been shown in a previous study by Ramirez-Molina et al 44, where majority of patients were stage 1b and tumors were all surgically excised. They demonstrated that patients with higher CHKα expression (1.91 times higher than that of healthy tissues) had a worse clinical outcome than those with low CHKα expression 44. We did not find any significant statistical correlation between [^18^F]D4-FCH and CHKα expression (*p* = 0.968). While this could be interpreted as a lack of influence of CHKα on substrate retention, our data disputes this hypothesis. a) Mathematical modelling supported a role for substrate trapping due to formation of phosphocholine and not transport, b) Voxel-by-voxel parametric maps indicated existence of high spatio-molecular heterogeneity particularly in 'CHKα negative' suggesting potential biopsy sampling challenges in these tumors might have contributed to this lack of correlation. A larger number of patients would have provided more confidence for this latter hypothesis. Nonetheless external validation in a larger lung cancer tissue bank demonstrated that this phenotype of variable CHKα protein expression in lung cancer was not only pertinent to our prospective study, with only ~50% of cases staining positive for the protein.

Following the study of Boellaard's lab, which employed pharmacokinetic modeling to validate the use of simplified methods for quantification of [^18^F]FCH 21, the correlation between SUV and kinetic parameters was also tested in order to establish whether simplified measures could be used instead of a full kinetic modelling. In agreement with Verwer et al. 21, we obtained poor correlations between both SUV and SUV_AUC_ and K_i__SA, K_i__CM and V_t__L. We obtained higher (but moderate) correlations between mean SUV and SUV_AUC_ evaluated at 60 min and K_i__P and K_1__SA. Our results showed that a simpler imaging protocol could not directly replace a full kinetic quantification as SUV does not take into account possible influences by metabolite formation or blood volume. However, the results showed that longer-scan SUV measures give higher correlations (60 min compared to 5 min) and supports SUV_60mean_ compared to SUV_60max_ 45.

This is an exploratory/ proof of concept study. Based on previous exploratory studies in our group, we estimated that 25 patients would be sufficient to provide initial estimates of the primary and secondary outcome measures. Over the 3-year period in running of this study, the limitations have been multifactorial. Both logistics and difficulties in enrollment have had an impact, with delay in completion of study and reaching target accrual. We recruited 17 patients, and all were included in the final analyses. Subsequently, data analysis was hampered with limitations in diagnostic tissue availability and the lack of [^18^F]FDG PET/CT in a few patients during staging, whereby no direct comparison with [^18^F]D4-FCH could be made.

Despite low [^18^F]D4-FCH uptake in NSCLC patients, there remains the question as to whether PET imaging using choline may still have utility in clinical practice. [^18^F]FDG PET/CT showed higher SUV than [^18^F]D4-FCH PET/CT , thus it is unlikely that [^18^F]D4-FCH will replace [^18^F]FDG for staging in lung cancer. However, FDG uptake is more homogeneous as it is utilized by all viable cells. We show here that [^18^F]D4-FCH uptake is heterogeneous, demonstrating potential use in treatment planning (Figure S3). The quantitative measurements provided by [^18^F]D4-FCH PET/CT uptake in this study may be complementary alongside [^18^F]FDG PET/CT in primary staging and management. Previous studies have explored the use of metabolic imaging to directly compare dose-escalation plans with standard uniform dose-prescriptions 46-48. Delivery of an integrated simultaneous boost guided by metabolic imaging may provide the spatial mapping sought in radiotherapy planning.

## Conclusion

In conclusion, we have shown late (60 min p.i) imaging with the new choline tracer, [^18^F]D4-FCH PET/CT designed according to the deuterium isotope effect principles, is feasible and that its intratumorally distribution is highly heterogeneous warranting future studies using this radiotracer for potential radiotherapy dose-delivery and treatment response monitoring.

## Supplementary Material

Supplementary figures and tables.

## Figures and Tables

**Table 1:** Patient characteristics.

Patient ID	Histology	TNM	Primary treatment
1	Adenocarcinoma	T4N0M1c	Palliative chemotherapy
2	Squamous	T4N2M1b	Chemotherapy, high dose palliative RT
3	Squamous	T2aN0M0	Surgery
4	Squamous	T1bN1M0	Surgery
5	Squamous	T4N1M0	Chemoradiation
6	Squamous	T2bN0M1b	Radical RT
7	Squamous	T1bN1M0	Surgery and adjuvant chemotherapy
8	Adenocarcinoma	T3N2M0	Chemoradiation
9	Adenocarcinoma	T1bN0M0	Surgery
10	Adenocarcinoma	T4N3M1a	Palliative chemotherapy
11	Squamous	T3N1M0	Surgery
12	Adenocarcinoma	T3N2M1b	Palliative chemotherapy
13	Adenocarcinoma	T3N1M1b	Chemotherapy
14	Squamous	T4N2M1a	Chemotherapy
15	Squamous	T3N2M1b	Chemotherapy
16	Adenocarcinoma	T4N2M0	Palliative RT
17	Adenocarcinoma	T3N2M1b	Chemotherapy

**RT** radiotherapy.

**Table 2:** Patient characteristics and PET parameters in primary tumor.

Patient	Sex	TNM	Histology	[^18^F]D4-FCH Dynamic Scan						[^18^F]D4-FCH Static Scan		[^18^F]FDG Static Scan	
				SUV 5 min		SUV 30 min		SUV 60 min		SUV 60 min		SUV 60 min	
				mean	max	mean	max	mean	max	mean	max	mean	max
1	F	T4N0M1c	Adenocarcinoma	4.51	7.88	4.76	8.42	4.77	8.46	4.73	8.64	No FDG	
2	F	T4N2M1b	Squamous	3.58	8.57	4.23	9.10	4.28	9.51	6.06	10.13	No FDG	
3	M	T2aN0M0	Squamous	2.28	2.65	2.64	3.01	2.80	3.17	2.95	6.14	7.46	11.95
4	F	T1bN1M0	Squamous	2.96	4.32	3.11	4.29	3.20	4.53	3.46	5.41	7.10	10.77
5	M	T4N1M0	Squamous	5.36	7.98	5.26	7.43	5.29	7.79	5.47	9.15	8.02	14.10
6	M	T2bN0M1b	Squamous	3.68	7.46	3.20	6.14	3.18	6.09	3.12	3.20	9.55	12.2
7	F	T1bN1M0	Squamous	2.85	5.32	2.59	3.87	2.54	3.83	2.24	3.18	4.96	6.89
8	F	T3N2M0	Adenocarcinoma	2.85	5.32	2.59	3.87	2.54	3.83			No FDG	
9	M	T1bN0M0	Adenocarcinoma	3.34	4.76	3.34	5.03	3.39	5.15	2.79	3.68	5.43	7.36
10	M	T4N3M1a	Adenocarcinoma	3.53	6.70	2.51	5.05	2.13	4.68	3.00	3.80	No FDG	
11	M	T3N1M0	Squamous	3.86	5.11	4.06	5.69	4.24	6.19	3.65	4.84	5.11	6.94
				4.23	7.04	4.68	7.43	4.84	8.15	4.82	6.99	11.93	22.64
12	F	T3N2M1b	Adenocarcinoma	2.61	2.61	2.23	2.23	2.08	2.08	1.94	2.87	3.70	5.63
13	F	T3N1M1b	Adenocarcinoma	4.18	4.62	3.54	4.17	3.23	4.57	2.77	4.45	5.53	10.01
14	F	T4N2M1a	Squamous	3.09	4.64	2.80	4.03	2.65	3.97	2.87	3.48	7.29	12.82
15	M	T3N2M1b	Squamous	2.41	5.38	3.28	6.33	3.41	6.39	3.54	7.59	4.11	7.58
16	M	T4N2M0	Adenocarcinoma	2.61	5.83	2.90	5.22	2.96	6.16	2.60	3.84	7.06	12.28
17	F	T3N2M1b	Adenocarcinoma	4.37	8.13	4.34	6.94	4.37	7.01			No FDG	

**M**, Male; **F**, Female

**Table 3:** ** Results of quantitative analyses of dynamic PET data.** Mean (± sd) values for Patlak K_i_ (K_i__P) [mL/cm^3^/min], SA K1 (K1_SA) [mL/cm^3^/min], SA K_i_ (K_i__SA) [mL/cm^3^/min], Logan V_t_ (V_t__L) [mL/cm^3^] and the compartmental model K_i_ (K_i__CM) [mL/cm^3^/min] in primary tumor and healthy lung. A double input SA was used to investigate the volumes of distribution of the parent and of the metabolite in the lesions. V_T_par_ and V_T_metab_ are the volumes of distribution for the parent and the metabolite, respectively. Parent and metabolite both contribute to the final PET signal.

			Single Input SA		Double Input SA		
Region	K_i__P	V_t__L	K_i__SA	K_1__SA	V_T_par_	V_T_metab_	K_i__SA
Primary tumor	0.05 (±0.04)	6.38 (±7.56)	8E-05 (±9E-05)	0.26 (±0.31)	0.99 (±0.70)	0.37 (±0.63)	8.75E-05 (±1E-04)
Healthy lung	0.0 2 (±0.22)	2.87 (±2.32)	4E-05 (±5E-05)	0.07 (±0.99)			

## Abbreviations

[^18^F]D4-FCH: [^18^F]fluoromethyl-(1,2-^2^H_4_) choline
[^18^F]FCH: [^18^F]fluoromethylcholine
[^18^F]FDG: [^18^F]fluorodeoxyglucose
[^18^F]FEC: [^18^F]fluoroethylcholine
1TCM2k: 1-tissue 2k compartmental model
2TCM4k: 2-tissue 3k reversible compartmental model
AIC: akaike information criterion
ARSAC: administration of radioactive substances advisory committee
CECT: contrast enhanced computed tomography
CHKα: choline kinase-α
CT: computed tomography
DI-SA: double input spectral analysis
EGFR: epidermal growth factor receptor
FMISO: [^18^F]fluoromisonidazole
HPLC: high-performance liquid chromatography
IRF: impulse response function
KRAS: kristen rat sarcoma viral oncogene
MHRA: medicine and healthcare products regulatory agency
NSCLC: non-small cell lung cancer
OSEM: ordered-subsets expectation maximization algorithm
PET: positron emission tomography
SA: spectral analysis
SUV: standardized uptake value
TMA: tissue microarrays
VOI: volumes of interest
