# Supplementary material for: Spatial heterogeneity of radiolabeled choline positron emission tomography in tumors of patients with non-small cell lung cancer: first-in-patient evaluation of [18F]fluoromethyl-(1,2-2H4)-choline
Source: Theranostics. 2020 Jul 9;10(19):8677–90. doi: 10.7150/thno.47298 (PMC7392021; doi:10.7150/thno.47298)
Supplement: Supplementary file 1 — Supplementary figures and tables. [file thnov10p8677s1.pdf]

SUPPLEMENTARY MATERIAL

Table S1 Patient characteristics and PET parameters.

Patient	Sex	TNM	Histology	Primary and metastases	[^{18}F]D4-FCH Dynamic SUV 5 min		Scan SUV 30 min		SUV 60 min		[^{18}F[D4-FCH Static Scan SUV 60 min		[^{18}F[FDG Static Scan SUV 60 min	
					**mean**	**max**	**mean**	**max**	**mean**	**max**	**mean**	**max**	**mean**	**max**
**1**	F	**T4N0M1c**	Adenocarcinoma	Primary tumor	4.51	7.88	4.76	8.42	4.77	8.46	4.73	8.64	**No FDG**	
				Satellite nodule							7.18	13.72		
				Scapular Met							7.17	10.64		
				Scalp Met							6.73	10.56		
				Liver Met							3.36	8.36		
**2**	F	**T4N2M1b**	Squamous	Primary tumor	3.58	8.57	4.23	9.10	4.28	9.51	6.06	10.13	**No FDG**	
				Hilar node							7.17	9.75		
				Liver Met							8.83	12.7		
**3**	M	**T2aN0M0**	Squamous	Primary tumor	2.28	2.65	2.64	3.01	2.80	3.17	2.95	6.14	7.46	11.95
				Paratracheal node*							3.42	5.11	2.38	2.82
				Hilar node*							3.01	4.32	2.04	2.19
**4**	F	**T1bN1M0**	Squamous	Primary tumor	2.96	4.32	3.11	4.29	3.20	4.53	3.46	5.41	7.10	10.77
				Hilar node¥							2.41	3.13	4.48	6.12
**5**	M	**T4N1M0**	Squamous	Primary tumor	5.36	7.98	5.26	7.43	5.29	7.79	5.47	9.15	8.02	14.10
**6**	M	**T2bN0M1b**	Squamous	Primary tumor	3.68	7.46	3.20	6.14	3.18	6.09	3.12	3.20	9.55	12.2
				L2 vertebral met									3.12	5.40
**7**	F	**T1bN1M0**	Squamous	Primary tumor	2.85	5.32	2.59	3.87	2.54	3.83	2.24	3.18	4.96	6.89
				Hilar node¥							2.64	3.46	3.10	3.83
**8**	F	**T3N2M0**	Adenocarcinoma	Primary tumor	6.11	12.48	6.70	14.30	6.93	16.19	4.26	8.61	**No FDG**	
				PT node							3.07	3.83		
**9**	M	**T1bN0M0**	Adenocarcinoma	Primary tumor	3.34	4.76	3.34	5.03	3.39	5.15	2.79	3.68	5.43	7.36
**10**	M	**T4N3M1a**	Adenocarcinoma	Primary tumor	3.53	6.70	2.51	5.05	2.13	4.68	3.00	3.80	**No FDG**	
				Hilar node							3.11	3.98		

				Hilar node							2.51	3.17		
				LUL tumor mass	3.01	7.10	2.61	3.80	2.47	3.97	2.29	3.10		
				PT node							2.66	3.73		
				Hilar node (2)							2.21	2.62		
				Hilar node (2)							2.82	3.30		
**11**	M	**T3N1M0**	Squamous	Primary tumor 1	3.86	5.11	4.06	5.69	4.24	6.19	3.65	4.84	5.11	6.94
				Primary tumor 2	4.23	7.04	4.68	7.43	4.84	8.15	4.82	6.99	11.93	22.64
**12**	F	**T3N2M1b**	Adenocarcinoma	Primary tumor (mix of pleural fluid)	2.61	2.61	2.23	2.23	2.08	2.08	1.94	2.87	3.70	5.63
**13**	F	**T3N1M1b**	Adenocarcinoma	Primary tumor (chest wall)	4.18	4.62	3.54	4.17	3.23	4.57	2.77	4.45	5.53	10.01
				Hilar node							3.22	4.77	4.87	10.53
				Hilar node							3.66	4.36	4.00	5.12
**14**	F	**T4N2M1a**	Squamous	Primary tumor	3.09	4.64	2.80	4.03	2.65	3.97	2.87	3.48	7.29	12.82
				Hilar node							2.12	2.89	2.16	2.95
				Ant rib met							3.73	4.22	6.10	10.54
				LUL tumor	1.49	3.40	1.56	2.25	1.55	2.35	2.19	2.69	4.28	5.25
**15**	M	**T3N2M1b**	Squamous	Primary tumor	2.41	5.38	3.28	6.33	3.41	6.39	3.54	7.59	4.11	7.58
				Hilar node							3.37	4.22	3.28	4.80
				Post Rib met							3.05	5.23	4.03	6.07
				Ant rib met							3.22	4.21	3.57	5.48
				Subcarinal node							2.55	4.45	5.33	8.90
**16**	M	**T4N2M0**	Adenocarcinoma	Primary tumor	2.61	5.83	2.90	5.22	2.96	6.16	2.60	3.84	7.06	12.28
				Hilar node							2.70	3.33	5.71	6.38
				Hilar node							1.86	2.33	7.24	10.56
				RLL tumor	3.67	7.53	4.39	7.59	4.16	7.10	3.86	5.47	9.46	14.91
				PT node							2.65	4.11	7.16	10.11
				PT node							3.34	4.48	8.18	13.64
**17**	F	**T3N2M1b**	Adenocarcinoma	Primary tumor	4.37	8.13	4.34	6.94	4.37	7.01	3.43	5.75	**No FDG**	
				PT node							3.26	4.54		
				Subcarinal node							2.24	2.73		

***** Node found to be negative at surgery

¥ Node found to be positive at surgery

M Male

F Female

PT Paratracheal lymph node

Met Metastasis

(TNM lung cancer staging AJCC 7TH Edition)

Table S2 Histogram analysis of parametric Ki_SA, K1_SA, Ki_P and Vt maps. Kurtosis, Skewness and Ratio (Skewness/Kurtosis) have been evaluated in tumor and healthy lung, for each patient. Last column shows CHKa expression in NSCLC. Last three rows show average (± sd) in n=10 healthy lung, n=3 lesions which showed negative CHKa expression, n=7 lesions which showed positive CHKa expression.

		Ki SA			K1 SA			Ki P			Vt			
		skewness	kurtosis	ratio	skewness	kurtosis	ratio	skewness	kurtosis	ratio	skewness	kurtosis	ratio	
**Pt 3**	*tum*	0.66	3.18	0.21	0.56	7.33	0.08	0.08	2.82	0.03	36.70	1566.00	0.02	**CHKa+**
	lung	0.64	2.85	0.22	0.44	2.16	0.20	1.05	3.61	0.30	26.65	911.23	0.03	
**Pt 4**	*tum*	4.07	25.39	0.16	1.41	4.41	0.32	1.05	3.61	0.29	19.08	385.51	0.05	**CHKa+**
	lung	-0.31	3.05	-0.10	0.41	2.78	0.15	-0.08	3.47	-0.02	13.37	231.13	0.06	
**Pt 5**	*tum*	10.56	149.13	0.07	3.03	14.68	0.21	-0.22	2.41	-0.09	30.02	1002.00	0.03	**CHKa+**
	lung	1.32	6.05	0.22	3.60	0.96	3.75	0.56	3.50	0.16	38.21	1656.00	0.02	
**Pt 6**	*tum*	1.22	5.59	0.22	2.19	18.34	0.12	0.50	3.18	0.16	24.83	661.36	0.04	**CHKa+**
	lung	1.26	5.99	0.21	1.05	3.18	0.33	1.15	4.34	0.26	19.92	579.28	0.03	
**Pt 7**	*tum*	5.07	31.99	0.16	2.88	13.90	0.21	-0.41	2.39	-0.17	16.55	344.84	0.05	**CHKa+**
	lung	3.58	25.22	0.14	2.54	17.05	0.15	2.07	7.17	0.29	35.30	1386.00	0.03	
**Pt 8**	*tum*	45.96	2175.00	0.02	2.85	29.19	0.10	0.77	5.05	0.15	56.56	3378.00	0.02	**CHKa+**
	lung	-0.08	3.28	-0.02	3.89	1.01	3.87	-0.55	3.23	-0.17	33.89	1355.00	0.03	
**Pt 9**	*tum*	0.46	2.28	0.20	1.13	3.78	0.30	0.20	2.30	0.09	4.04	24.77	0.16	**CHKa+**
	lung	4.33	24.21	0.18	1.67	5.69	0.29	0.51	1.50	0.34	3.55	17.94	0.20	
**Pt 10**	*tum*	0.03	1.98	0.01	0.74	2.54	0.29	0.05	2.72	0.01	10.46	130.21	0.08	**CHKa-**
	lung	0.91	3.69	0.25	1.67	5.22	0.32	0.42	2.44	0.17	29.12	861.07	0.03	
**Pt 12**	*tum*	0.58	2.66	0.22	-0.72	2.63	-0.27	-0.23	2.29	-0.10	7.59	76.27	0.10	**CHKa-**
	lung	0.30	2.14	0.14	3.75	18.16	0.21	0.19	2.73	0.07	0.38	8.78	0.04	
**Pt 2**	*tum*	1.12	4.08	0.27	1.26	4.19	0.30	0.18	1.77	0.10	45.46	2279.00	0.02	**CHKa-**
	lung	1.93	15.19	0.13	1.42	5.78	0.25	1.37	6.82	0.20	20.84	545.00	0.04	
**Average ± SD**	**Lung [n = 10]**	1.39 ± 1.52	9.17 ± 9.01	0.14 ± 0.11	2.05 ± 0.33	6.20 ± 6.27	0.95 ± 1.51	0.67 ± 0.76	3.88 ± 1.81	0.16 ± 0.16	22.13 ± 13.08	755.14 ± 582.63	0.05 ± 0.05	
	**Tum_CHKa- [n = 3]**	0.58 ± 0.55	2.91 ± 1.07	0.17 ± 0.14	0.43 ± 1.02	3.12 ± 0.93	0.11 ± 0.33	-0.01 ± 0.21	2.26 ± 0.47	0.01 ± 0.10	21.17 ± 21.08	828.50 ± 1256.46	0.07 ± 0.04	
	**Tum_CHKa+ [n = 7]**	9.71 ± 16.37	341.79 ± 810.02	0.15 ± 0.08	2.01 ± 0.98	13.06 ± 8.99	0.19 ± 0.10	0.28 ± 0.53	3.11 ± 0.98	0.06 ± 0.16	26.83 ± 16.74	1051.78 ± 1142.30	0.05 ± 0.05	

Table S3 Comparison of simplified parameters to K_i_P, K_1_SA, K_i_SA, V_t_L and K_i_CM. Results of the linear regression analysis.

N=62	Simplified parameter	K_i_P			K_1_SA			K_i_SA			V_t_L			K_i_CM		
		slope	intercept	R_2	slope	intercept	R_2	slope	intercept	R_2	slope	intercept	R_2	slope	intercept	R_2
**[^{18}F]D4-FCH**	**SUV$_{5mean}$**	0.02	-0.02	0.50	0.06	-0.01	0.20	-4E-07	5E-06	5E-03	0.05	0.03	0.09	3.27	-3.36	0.47
	**SUV$_{60mean}$**	0.02	-0.02	0.53	0.09	-0.10	0.46	2E-07	6E-06	1E-03	0.07	-0.10	0.21	3.23	-3.46	0.49
	**SUV$_{5max}$**	3E-04	0.04	5E-04	-3E-04	0.16	4E-03	1E-07	1E-05	0.09	-6E-04	0.13	0.01	6E-03	5.40	1E-03
	**SUV$_{60max}$**	1E-03	0.03	0.13	1E-03	0.13	0.02	-1E-07	9E-06	0.04	7E-04	0.09	1E-03	0.18	3.13	0.16
	**SUV$_{AUC_5mean}$**	0.04	-0.01	0.39	0.08	0.05	0.10	2E-06	1E-05	0.04	0.04	0.05	0.02	0.02	5.23	4E-03
	**SUV$_{AUC_60mean}$**	4E-04	-0.02	0.52	1E-03	-0.10	0.45	-1E-07	5E-06	0.03	1E-03	-0.10	0.20	0.05	-3.60	0.49
	**SUV$_{AUC_5max}$**	2E-04	0.04	0.01	-6E-04	0.16	3E-03	1E-08	5E-06	0.01	-1E-03	0.12	0.01	3E-03	3.06	0.14
	**SUV$_{AUC_60max}$**	2E-05	0.03	0.12	3E-04	0.13	0.02	-1E-09	8E-06	0.02	5E-06	0.10	4E-04	3E-03	3.06	0.14

A

Time (min)	% parent fraction (mean ± sd)	% total metabolite (mean ± sd)
5	0.98 ± 0.01	0.02 ± 0.01
10	0.95 ± 0.03	0.05 ± 0.03
15	0.83 ± 0.10	0.17 ± 0.10
30	0.60 ± 0.18	0.40 ± 0.18
60	0.48 ± 0.22	0.52 ± 0.22

B

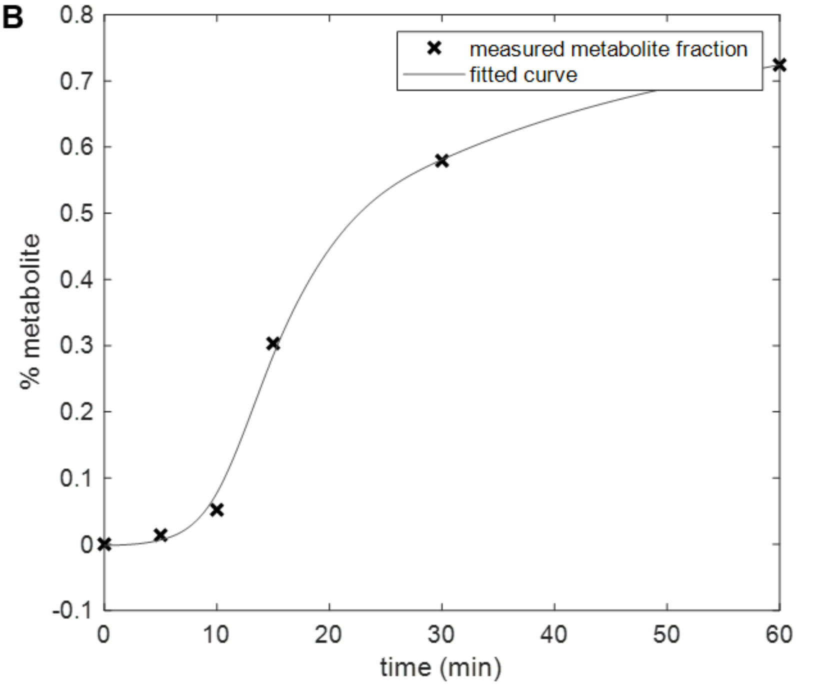

Figure S1 Parent and metabolite fractions measured by HPLC. A) Summary of the fraction of parent tracer and metabolite (mean ± standard deviation) measured by HPLC for each patient ($N = 11$). B) Typical fit of the metabolite fraction data for D4 choline.

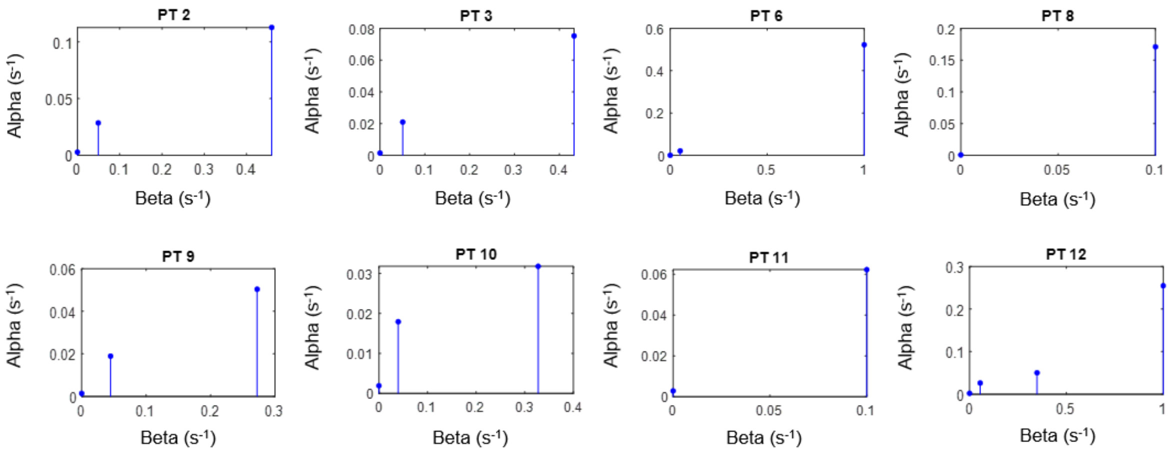

Figure S2 Spectral Analysis results. The panel shows the spectra of 8 patients where an irreversible kinetic component was found (in 0). The x axis represents the amplitude of the kinetic components while the position in the spectrum (y axis) reflects the nature of the exchanges between different compartments.

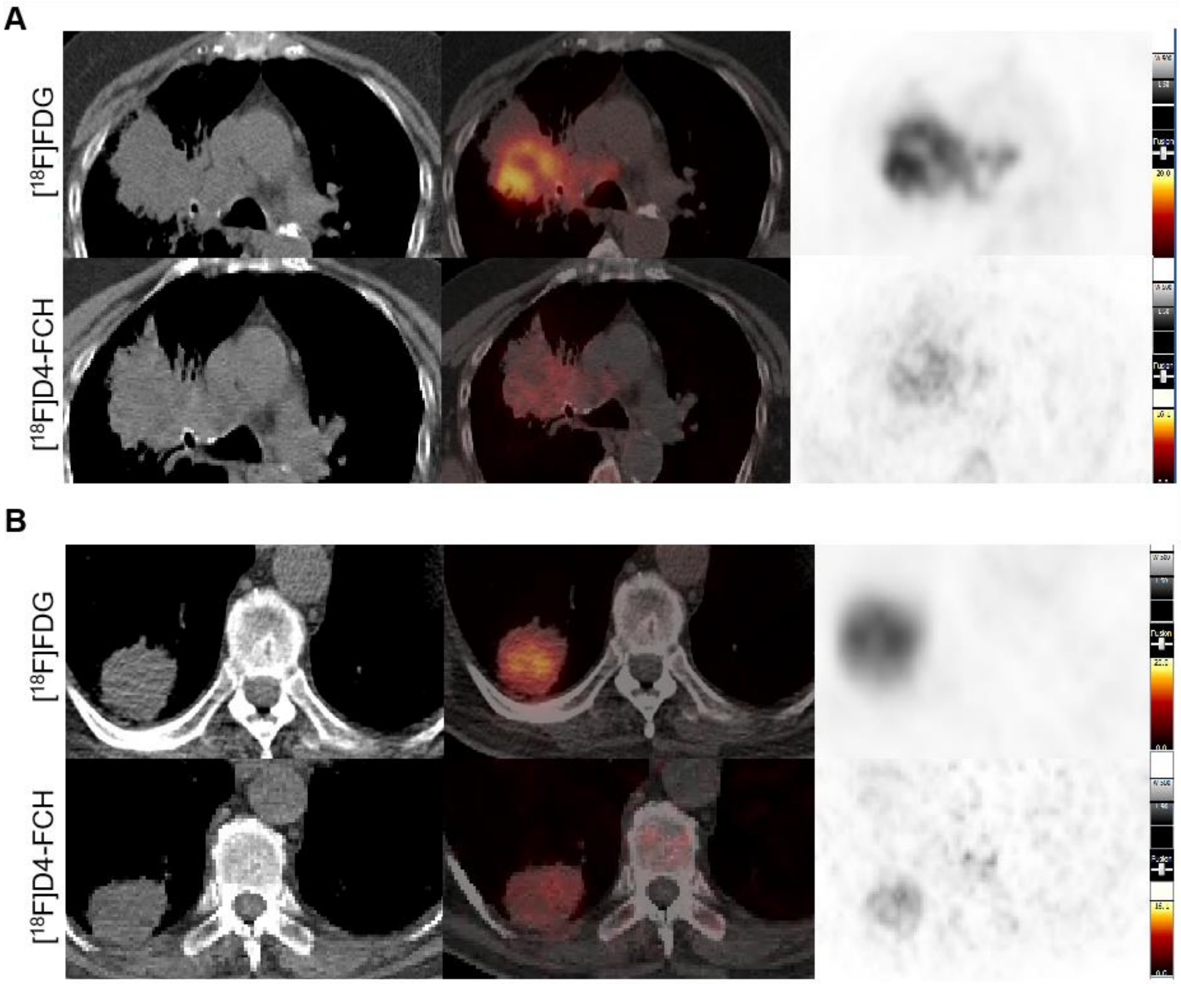

Figure S3 Display of [^{18}F]D4-FCH and [^{18}F]FDG images at different intensities to illustrate tumoral heterogeneity. Panel **A** and **B** show uptake of [^{18}F]FDG and [^{18}F]D4-FCH in tumors of patient's 16 and 9, respectively. Because, sufficient tumor variations in radiotracer localization was not visualized at SUV 0-10 (often used clinically for display) the highest display is set to 20 in the case of [^{18}F]FDG images and 16 for [^{18}F]D4-FCH images. There are visually non-overlapping and more heterogeneous intensity localization in [^{18}F]D4-FCH derived images even at this display level.
